# Influence of Slow-Paced Breathing on Inhibition After Physical Exertion

**DOI:** 10.3389/fpsyg.2019.01923

**Published:** 2019-08-22

**Authors:** Sylvain Laborde, Theresa Lentes, Thomas J. Hosang, Uirassu Borges, Emma Mosley, Fabrice Dosseville

**Affiliations:** ^1^Department of Performance Psychology, German Sport University Cologne, Cologne, Germany; ^2^Normandie Université, UFR STAPS, EA 4260, Cesams, Caen, France; ^3^Experimental Psychology Unit, Department of Psychology, Helmut Schmidt University, Hamburg, Germany; ^4^Southampton Solent University, Southampton, United Kingdom; ^5^Normandie Université, UMR-S 1075 COMETE, Caen, France; ^6^INSERM, UMR-S 1075 COMETE, Caen, France

**Keywords:** acute exercise, physical activity, executive function, cognition, heart rate variability

## Abstract

This research aims to investigate whether slow-paced breathing (SPB) improves adaptation to psychological stress, and specifically inhibition, when it is performed before or after physical exertion (PE). According to the resonance model, SPB is expected to increase cardiac vagal activity (CVA). Further, according to the neurovisceral integration model, CVA is positively linked to executive cognitive performance, and would thus play a role in the adaptation to psychological stress. We hypothesized that SPB, in comparison to a control condition, will induce a better adaptation to psychological stress, measured via better inhibitory performance. Two within-subject experiments were conducted with athletes: in the first experiment (*N* = 60) SPB (or control – neutral TV documentary) was realized before PE (“relax before PE”), and in the second experiment (*N* = 60) SPB (or the watching TV control) was realized after PE (“relax after PE”). PE consisted of 5 min Burpees, a physical exercise involving the whole body. In both experiments the adaptation to psychological stress was investigated with a Stroop task, a measure of inhibition, which followed PE. Perceived stress increased during PE (partial η^2^ = 0.63) and during the Stroop task (partial η^2^ = 0.08), and decreased during relaxation (partial η^2^ = 0.15), however, no effect of condition was found. At the physiological level PE significantly increased HR, RF, and decreased CVA [operationalized in this research via the root mean square of successive differences (RMSSD)] in both experiments. Further, the number of errors in the incongruent category (Stroop interference accuracy) was found to be lower in the SPB condition in comparison to the control condition, however, these results were not mediated by RMSSD. Additionally, the Stroop interference [reaction times (RTs)] was found to be lower overall in “relax before PE,” however, no effect was found regarding SPB and Stroop interference (RTs). Overall, our results suggest that SPB realized before or after PE has a positive effect regarding adaptation to psychological stress and specifically inhibition, however, the underlying mechanisms require further investigation.

## Introduction

Executive functions underpin goal-directed behavior and are essential for self-control (Miyake et al., 2000; Diamond, 2013; Kotabe and Hofmann, 2015). Executive functions may be hindered by factors such as stress (e.g., Arnsten, 2009), fatigue (Kurzban et al., 2013; Inzlicht et al., 2014; Schmit and Brisswalter, 2018), and pressure (e.g., Laborde et al., 2014). The aim of this paper is to investigate the influence of a relaxation method [slow-paced breathing (SPB)], to prevent inhibition failure during psychological stress following physical exertion (PE).

Several neurological mechanisms have been identified in the literature to explain the influence of perceived stress (PS; e.g., Archer et al., 2018), mental fatigue (e.g., Guo et al., 2018) or physical fatigue (e.g., Tanaka et al., 2013) on inhibition failure. In this research, we focus on the role of the autonomic nervous system, and more specifically of its parasympathetic branch. The neurovisceral integration model (Thayer et al., 2009; Smith et al., 2017) assumes that similar structures are involved in the regulation of executive performance and cardiac functioning. The functional organization of these structures is depicted by the central autonomic network (Benarroch, 1993), the output of this network being cardiac vagal activity (CVA), the activity of the vagus nerve which regulates cardiac functioning (Thayer et al., 2009; Laborde et al., 2017b). Based on this functional organization, the neurovisceral integration model (Thayer et al., 2009; Smith et al., 2017) assumes that the effectiveness of the central autonomic network is reflected in CVA and can be indexed via heart rate variability (HRV), the time interval between adjacent heartbeats (Malik, 1996; Berntson et al., 1997). Specifically, the neurovisceral integration model assumes that a higher resting CVA is linked to better executive functioning (Wendt et al., 2015; Albinet et al., 2016; Spangler and Friedman, 2017; Spangler et al., 2018). In this paper, we operationalize CVA with the RMSSD (Malik, 1996; Berntson et al., 1997), which has been found to be less affected by respiratory influences than other HRV variables suggested to index CVA (Hill et al., 2009). Given that CVA reflects self-regulation and higher levels of CVA promote better executive performance (Thayer et al., 2009), it serves as a valuable measure when assessing performance under both physiological and psychological stress.

Psychological stress occurs when an individual perceives that personal or environmental demands tax or exceed his or her adaptive abilities (Lazarus and Folkman, 1984). Even if psychological stress can be considered as an idiosyncratic phenomena, given it will differ across individuals and situations, a common method of inducing stress is using tasks taxing executive functions. In addition, putting an emphasis on performing well helps to represent a situation which differs markedly from resting states in terms of psychological demands placed on the individual. For example, the Color Word Stroop Test (CWST; Stroop, 1935), a classical cognitive test to investigate inhibition (Diamond, 2013), has been used to create psychological stress (e.g., Vazan et al., 2017), and was found to increase biological markers of stress (e.g., Brugnera et al., 2018). In the CWST, an inhibitory interference occurs when the processing of a stimulus feature affects the simultaneous processing of another attribute of the same stimulus (Stroop, 1935). Many variations exist, but the basic experimental paradigm depicted in the CWST is the use of color words printed either in the same color for the congruent category (for example, “BLUE” printed in blue color) or in a different color for the incongruent category (for example, “BLUE” printed in red). The participant has then to name the color in which the word is printed. The accuracy (number of errors) and reaction times (RTs) are measured to investigate the Stroop test performance (Stroop, 1935; Scarpina and Tagini, 2017). Better performance in this test reflects better inhibitory control which directly reflects the processing of incongruent stimuli in comparison to congruent stimuli. This differential processing constitutes the basis of the so-called Stroop interference, although a high heterogeneity to calculate the Stroop interference is reported in the literature (Scarpina and Tagini, 2017). Inhibition is primarily displayed by the error rate (accuracy) (McDowd et al., 1995), which represents as well an index of the ability to maintain the task’s goal temporarily in a highly retrievable state (Kane and Engle, 2003). Assessing RTs may also be useful to uncover other processes linked to inhibition (Scarpina and Tagini, 2017).

The CWST and its variations (which we refer to thereafter as “Stroop tests”) have already been investigated with HRV (Hoshikawa and Yamamoto, 1997; Prinsloo et al., 2011; Satish et al., 2015; Subramanya and Telles, 2015; Vazan et al., 2017; Zeki Al Hazzouri et al., 2017), however, only limited studies exist examining Stroop performance related to CVA (Johnsen et al., 2003; Subramanya and Telles, 2015; Albinet et al., 2016). In several studies, the Stroop test has been used with the mere purpose to create psychological stress (Hoshikawa and Yamamoto, 1997; Satish et al., 2015; Vazan et al., 2017), and no link with inhibition performance has been established. In other studies, although Stroop performance has been investigated with HRV, it has not been directly related to CVA indices (Prinsloo et al., 2011; Zeki Al Hazzouri et al., 2017). Among the studies linking Stroop performance and CVA, Johnsen et al. (2003) found that patients with dental fear having a higher resting CVA had shorter RTs to the incongruent color words and to the threat words compared to patients with lower resting CVA. However, Stroop interference accuracy (number of errors) was not measured, which does not provide a holistic representation of inhibition (McDowd et al., 1995). Albinet et al. (2016) showed that CVA improvements linked to a 5-months aquaerobics training program in older adults were linked to improvements on the Stroop interference accuracy (lower number of errors). Those two studies would be in line with the assumptions of the neurovisceral integration model (Thayer et al., 2009). On the contrary, Subramanya and Telles (2015) found that Stroop performance was better (less errors in the incongruent condition) with a decrease in high-frequency HRV, which usually depicts CVA when breathing patterns are comprised between 9 and 24 (Berntson et al., 1997), and increases in low-frequency HRV. However, these results were observed following a cyclic meditation condition involving slow body movements and slow breathing patterns, so there may have been some confounding effects of the experimental manipulation, and we can also speculate that in this case low-frequency HRV may have been mainly vagally driven. In summary, limited studies have linked Stroop performance to CVA, investigating either only RTs or accuracy (number of errors). Our research aims to address this gap and link CVA to both Stroop performance RTs and accuracy (number of errors).

When considering the effects of PE on Stroop performance, one should distinguish the timing of the Stroop test. During exercise cognitive performance is usually impaired and improves after exercise (Lambourne and Tomporowski, 2010), unless exhaustion is reached (Schmit et al., 2015). After acute exercise, RT on the Stroop interference is shorter after PE in comparison to a resting control condition (Alves et al., 2012), or in comparison to before PE (Johnson et al., 2016), regardless of the fitness level of the participants (Chang et al., 2014). Improvements on the Stroop interference accuracy (number of correct answers) after acute exercise were found (Peruyero et al., 2017), however, some mixed findings were also reported (Vincent and Hall, 2017). Improved cognitive performance after acute exercise is usually linked to an increase in physiological arousal (Chang et al., 2014). In line with the inverted-U hypothesis, moderate arousal is linked to optimal cognitive performance, while if arousal levels are either too low or too high cognitive performance is impaired (Ploughman, 2008; Kashihara et al., 2009). Acute effects of physical activity on Stroop performance are explained in particular via increased brain-derived neurotrophic factor (Griffin et al., 2011) and brain activation in the left dorsolateral prefrontal cortex (Yanagisawa et al., 2010), but do not seem to be related to changes in cerebral blood flow (Ogoh et al., 2014). At present, we are not aware of any research linking acute physical fatigue, Stroop performance, and CVA, and our research aims to address this gap.

Physical exertion will induce a drop in CVA, due to the activation of the sympathetic nervous system and the inhibition of the parasympathetic nervous system (Goldsmith et al., 2000; Iellamo, 2001; Winsley, 2002; Aubert et al., 2003; Stanley et al., 2013; Michael et al., 2017). Stopping PE induces a parasympathetic reactivation, which speed and magnitude depends on the fitness level of the individual (Stanley et al., 2013; Romero et al., 2017). If we follow the assumptions of the neurovisceral integration model (Thayer et al., 2009), the fact cognitive performance is decreased during exercise but improved after exercise (Lambourne and Tomporowski, 2010) may be linked to the parasympathetic deactivation observed during PE and reactivation after PE. Importantly, it may be possible to influence the speed and magnitude of CVA recovery after PE via specific strategies. Indeed, many factors were found to influence CVA (Laborde et al., 2018b), and some of them will be particularly adapted for athletes (Laborde et al., 2018c). Among those, we focus in this research on SPB.

Slow-paced breathing is a breathing technique with controlled inhalation and exhalation times (“paced”), realized at a slower pace, around 6 cycles per minute (cpm) than spontaneous breathing, which is usually comprised between 12 and 20 cpm in adults (Sherwood, 2006; Tortora and Derrickson, 2014). The pacing is usually realized via a visual, audio, or kinesthetic pacer (e.g., Allen and Friedman, 2012). According to the resonance model (Lehrer and Gevirtz, 2014), four processes play a role to understand the effects of SPB at 6 cpm: (1) the phase relationship between heart rate (HR) oscillations and breathing at 6 cpm; (2) the phase relationship between HR and blood pressure oscillations at 6 cpm; (3) the activity of the baroreflex; and (4) the resonance characteristics of the cardiovascular system. Combined, those processes are expected to strengthen homeostasis in the baroreceptor (Vaschillo et al., 2002, 2006; Lehrer et al., 2006), which results in improving gas exchanges at the level of alveoli and in increasing vagal afferences (Lehrer and Gevirtz, 2014). Evidence has already been found for both acute (Laborde et al., 2017a) and chronic (Laborde et al., 2019) increases in CVA (i.e., vagal efferent activity) following SPB interventions.

Slow-paced breathing has already been shown to improve cognitive functioning, with inhibition and working memory (Prinsloo et al., 2011). Prinsloo et al. (2011) investigated a modified Stroop test, combining the classical inhibition component (i.e., naming the ink in which a word corresponding to another color is printed) to a working memory component, asking participants to remember how many control white squares appeared on the screen. Participants were allocated either to a SPB condition with biofeedback (seeing live the effects of SPB on their HRV via a device) or to a control condition where they were breathing spontaneously, for 10 min. Results showed no differences between conditions on the Stroop test inhibition component (number of errors), however, the working memory performance of the SPB group was better when compared to the control group. Limitations of this study were a reduced sample size (*N* = 18 in a between-subjects design), the fact that inhibition and working memory were mixed in the modified Stroop test, which did not allow for drawing clear conclusions about the specific executive functions targeted, and finally the link between Stroop performance and CVA was not investigated. To conclude, research investigating the effects of SPB on inhibition is still required.

In summary, based on the research gaps identified in the literature, the aim of this research was to investigate the influence of a short-term SPB technique (in comparison to a watching TV control condition) on adaptation to psychological stress characterized via inhibition performance, before (Experiment 1) and after (Experiment 2) PE. The overall research was conceived as a mixed-model design, both experiments were conducted as within-subject designs (i.e., meaning participants take part in both the SPB and the control conditions of the same experiment), however, participants of Experiment 1 were not participating to Experiment 2, corresponding to the between-subject part. The within-subject part is always recommended in experiments involving HRV to limit inter-individual differences (Quintana and Heathers, 2014; Laborde et al., 2017b), however, given participating in four experimental sessions may have created some habituation effect, we split our research in two experiments, so that each participant was participating to two experimental sessions. At the subjective level, we expected PE and the CWST to increase the level of PS given both activities may lead the individual to perceive that demands of the task tax or exceed current adaptive abilities, while the relaxation moment would decrease the level of PS (Lazarus and Folkman, 1984; Lehrer and Gevirtz, 2014; Conway and Rubin, 2016). Further, we expected the SPB condition to show larger effect sizes than the control condition, based on its relaxing effects (Lehrer and Gevirtz, 2014). Further, at the physiological level, we expect PE to increase HR and RF and decrease RMSSD, reflecting cardiovascular and respiratory adaptations to acute exercise (Stanley et al., 2013). Regarding our main hypothesis, based on the neurovisceral integration model (Thayer et al., 2009) and on the resonance model (Lehrer and Gevirtz, 2014), we hypothesize that in both experiments, SPB will improve inhibition (Stroop interference) in terms of accuracy (number of errors) and RTs. The improvement in Stroop interference will be mediated by resting RMSSD before starting the Stroop test.

## Materials and Methods

### Participants

In order to determine our sample size, we utilized previous research combining SPB and inhibition (Prinsloo et al., 2011). The study of Prinsloo et al. (2011) did not find any effect of SPB on inhibition and was likely underpowered (*N* = 18 for a between-subject design).^1^ Based on this previous work, we computed an *a priori* power analysis with G^∗^Power (Faul et al., 2007) based on a small effect size, for a repeated-measures ANOVA with a within-between interaction, with a power of 0.80 and an alpha level of 0.05, which gave us a total sample size of 120. A total of *N* = 60 participants (35 men, 25 women, mean age = 25.57 years old, age range = 19–40, BMI: *M* = 23.62, *SD* = 2.52) took part in Experiment 1, where the relaxation condition (SPB vs. watching TV control) was realized before physical PE. We refer thereafter to this first experiment as “relax before PE.” Similarly, a total of *N* = 60 participants took part in Experiment 2 (38 men, 22 women, Mean age = 24.87 years old, age range = 18–41, BMI: *M* = 22.86, *SD* = 2.29), where the relaxation condition (SPB vs. watching TV control) was realized after PE. We refer thereafter to this second experiment as “relax after PE.” Exclusion criteria were self-reported cardiovascular diseases, and other chronic diseases that might influence breathing or HR patterns, such as asthma, diabetes, psychiatric, and neurological diseases (Laborde et al., 2017b) or being color-blind, making them ineligible to complete the CWST. Participants were students at the German Sport University. The protocol of the study was approved by the Ethics committee of the German Sport University (N° 175/2016).

### Material and Measures

#### Cardiac Vagal Activity

Cardiac vagal activity was operationalized via HRV and more specifically with RMSSD. An electrocardiography (ECG) device (Faros 180°, Mega Electronics, Kuopio, Finland) was used during the experiment to assess HRV, with a sampling rate of 500 Hz. We used two disposable ECG pre-gelled electrodes (Ambu L-00-S/25, Ambu GmbH, Bad Nauheim, Germany). The negative electrode was placed in the right infraclavicular fossa (just below the right clavicle) while the positive electrode was placed on the left side of the chest, below the pectoral muscle in the left anterior axillary line. The Faros device was taped to the participants’ chest in order to avoid moving too much while the participant was performing PE. From ECG recordings we extracted RMSSD with Kubios^©^ (University of Eastern Finland, Kuopio, Finland). The full ECG recording was inspected visually, and artifacts were corrected manually (Laborde et al., 2017b). As recommended by Laborde et al. (2017b), respiratory frequency (RF) was also taken into account. RF was computed via the ECG derived respiration algorithm of Kubios^©^ (Tarvainen et al., 2014).

#### Perceived Stress

A visual analog scale (VAS) was used to measure PS (Lesage and Berjot, 2011). Participants were asked “How stressed do you feel right now?” and they responded by marking a cross on a 100 mm line with two anchors (“not at all stressed” to “very much stressed”).

#### Physical Exertion – Burpees

Physical exertion was achieved with a modified version of the Burpee test (Podstawski et al., 2013). The Burpee test was named after the American physiologist Royal H. Burpee (1940), and was originally designed to measure agility and coordination. Burpees are physical exercises involving the whole body and requires no additional equipment. The following version of the Burpee was performed,^2^ with these instructions: (1) start from a standing position; (2) bend over and place both hands firmly on the ground in front of the feet; (3) kick (or step) both feet back into a push-up position and lower the entire body to the ground (this is not a push-up); (4) the chest and thighs need to make full contact with the ground; (5) then extend the arms, lifting the chest and jump (or step) both feet in toward the chest; and (6) stand, jump (opening the hips fully), and clap hands behind the head while in the air.

#### Slow-Paced Breathing

Similar to previous research (Laborde et al., 2017a), the SPB exercise was conducted with a video showing a little ball moving up and down at the rate of 6 cpm. Participants having to inhale continuously through the nose while the ball was going up, and exhale continuously with pursed lips when the ball was going down. The video used was the same used in Laborde et al. (2017a), displaying a 3 × 5-min SPB exercise, with a 1-min break between each unit, corresponding to a total of 17-min. Exhalation (5.5 s) was slightly longer than inhalation (4.5 s), because a prolonged exhalation contributed to larger beat-to-beat heart fluctuations compared to prolonged inhalation, and therefore induce higher CVA (Strauss-Blasche et al., 2000).

#### TV Neutral Documentary

The control condition (CON) used a TV documentary about world travel destinations, this was shown to the participants for the same duration as the SPB exercise (17 min). This TV documentary was found to be subjectively emotionally neutral in a previous pilot study.

#### Inhibition Performance (Measured With the Stroop Interference)

We used the computerized version of the CWST with verbal responding available in the Inquisit library,^3^ and ran this with the Inquisit software (Inquisit 5 [Computer Software], 2016). We used a 15-in. flat-screen monitor (1,280 × 960 pixels at 60 Hz) at a viewing distance of 60 cm. Words appeared in 28-pt Arial font in the middle of a white screen. Three types of stimuli were used: colored square (congruent control stimuli), colored words displayed with the color corresponding to the word (congruent stimuli, for example the word “green” is displayed in the color green), and colored words displayed with an inconsistent color (incongruent stimuli, for example the word “green” is displayed in the color red). Participants were asked to name the color in which the word was written as fast and as accurately as possible, while ignoring the written meaning of the word. A headset mounted microphone recorded the verbal answers. A familiarization was realized with 20 trials. For the test, participants completed 84 trials (4 colors – red, green, blue, black) × 3 color stimulus congruency (congruent, incongruent, control squares) × 7 repetitions = 84 trials. Stimuli stayed on screen until response, latencies were measured from onset of stimuli. The intertrial interval was of 200 ms, and the error feedback (a red cross) of 400 ms.

#### Procedure

**FIGURE 1:**
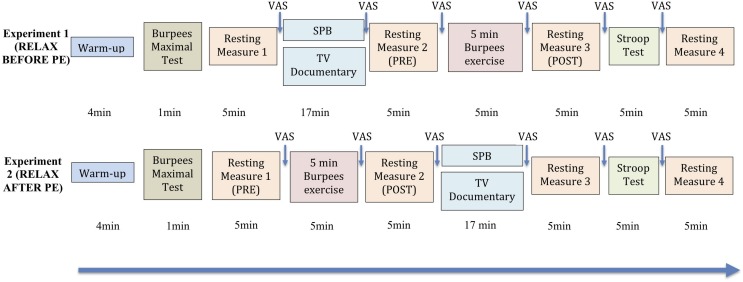
Experimental protocol. “Relax before PE”: experiment in which the relaxation moment (either slow-paced breathing or watching the TV documentary) took place before the 5 min Burpees exercise; “Relax after PE”: experiment in which the relaxation moment (either slow-paced breathing or watching the TV documentary) took place after the 5 min Burpees exercise. For the manipulation check of the Burpee exercise, “PRE” refers to the 5 min resting measure realized before PE (so resting measure 1 for “relax before PE” and resting measure 2 for “relax after PE”), while “POST” refers to the 5 min resting measure realized after PE (so resting measure 2 for “relax before PE” and resting measure 3 for “relax after PE”). VAS: Visual Analog Scale (perceived stress).

Participants were recruited via flyers on the campus of the local University and via posts on social networks groups linked to the local University. For each experiment, there were two testing sessions involved (lasting around 90 min each, see Figure 1 for full description). The experimental order of the sessions was counterbalanced. The two sessions were separated by 1 week, to keep learning effects to a minimum, and took place at the same time of the day, given this parameter may influence HRV (van Eekelen et al., 2004) and performance (Folkard, 1990; Laborde et al., 2018a). Participants were either taking part in Experiment 1 or Experiment 2, they could not participate to both. They were asked to wear sports clothes to take part in the experiment. Prior to the testing sessions, participants were instructed not to drink or eat anything but water during the 2 h before the experiment, or take part in any strenuous exercise or drink alcohol for the 24 h prior testing (Laborde et al., 2017b). Both experiments were the same in their conception, the only aspect that differed was whether the relaxation moment was taking place before (Experiment 1) or after (Experiment 2) PE.

Upon arrival to the laboratory, participants were asked to fill out an informed consent form and a demographic questionnaire regarding variables potentially influencing HRV (Laborde et al., 2017b). At the beginning of the SPB condition, participants also received a short video introduction on how to perform the technique correctly, which was checked by the experimenter. Then participants were asked to perform a warm up^4^ for 4 min involving squats, lunges, leg swings, jumping jacks, press up’s, and squat thrusts. The warm up was chosen in agreement with an expert strength and conditioning coach from the German Sport University who was part of the research team. To prepare the PE (5 min Burpees exercise), participants had to perform five trials Burpees, so that the experimenter could check whether they were performed correctly. Then participants had to do a maximal Burpees test for 1 min, with the instruction to perform as many technically correct Burpees as they could. We then calculated 70% of this maximal number, and this gave the number of Burpees for the participants to realize during the 5 min Burpees exercise. For example, if the participant performed a maximal number of 20 Burpees, 70% × 20 = 14, for the 5 min Burpees exercise he/she would have then to perform 14 Burpees each minute for 5 min. During the 5 min Burpees exercise, the realization of the Burpees was paced by the experimenter, giving a signal as a time marker to perform each Burpee, so the participant could better distribute his/her effort along the 5 min. The value of 70% was chosen based on a pilot study realized with 10 participants, where 70% was found to be an achievable compromise between achievability and degree of exhaustion, which had to be higher than 17 on the Borg Scale of Perceived Exertion (Borg, 1982). The 1 min Burpees maximal test was realized at the beginning of each session, given we wanted to account for potential differences in daily fitness level. The number of Burpees to be realized during the 5 min Burpees exercise was then based on the maximal number of Burpees achieved during the 1 min Burpees test at the beginning of the same session.

The relaxation task (either SPB or watching TV control) was placed either before (Experiment 1) or after (Experiment 2) PE. Finally participants had to perform the Stroop test, that lasted between 4 and 5 min. Between each block of the sessions, a HRV resting measure of 5 min was taken, based on the Task Force recommendation (Malik, 1996). HR and RF were derived from this HRV measurement. The HRV resting measure was realized in a sitting position with eyes closed, knees at 90°, hands on the thighs. At the end of the second testing session, participants were debriefed and thanked.

### Data Analysis

Due to technical problems, the ECG data of the last 13 participants of Experiment 2 were lost, therefore the sample size of Experiment 2 was reduced to *N* = 47. Regarding the HRV data, RMSSD was extracted from the Kubios output. RF (respiratory cycles per minute) was calculated multiplying the EDR (ECG derived respiration) value obtained via the Kubios algorithm by 60.

For the Stroop test, the number of incorrect answers was retrieved for the congruent colored squares, as well as for the congruent and incongruent stimuli. Regarding response times, we analyzed only those of the correct answers. Then we used two filters (see Lautenbach et al., 2016). In the first filter, trials with response times lower than 200 ms and higher than 3000 ms were excluded in order to account for extreme results (see Putman and Berling, 2011). The second filter then screened for RTs higher or lower than two standard deviations from the mean, which were also removed to account for outliers (see Dresler et al., 2009).

The VAS data and the Stroop performance data were normally distributed and homoscedastic. The physiological data (HRV, HR, RF) were not normally distributed, thus a log-transform (Log 10) was used to achieve normal distribution (Laborde et al., 2017b), and data were homoscedastic. Regarding the physiological data, we ran the analyses with the log-transformed values, however, we indicate as descriptive values the raw data, given they make more sense for the reader. For RMSSD, we controlled as well for the influence of covariates that have been linked to variations in CVA, such as RF, age, gender, smoking status, and BMI.

For the manipulation check related to PS, we ran three successive repeated-measures ANOVA for the relaxation technique, the 5 min Burpees exercise, and the Stroop task. We had time (before vs. after), condition (SPB vs. CON) as within-subject independent variable, and relaxation moment (“relax before PE” or “relax after PE”) as between-subject independent variable.

For the physiological manipulation check related to the 5 min Burpees exercise, “PRE” refers to the 5 min resting measure realized before PE (so resting measure 1 for “relax before PE” and resting measure 2 for “relax after PE”), while “POST” refers to the 5 min resting measure realized after PE (so resting measure 2 for “relax before PE” and resting measure 3 for “relax after PE”). As manipulation check, we wanted to ensure that PE was leading to physiological changes usually seen with acute exercise, meaning we expected a main effect of time (“PRE PE” vs. “POST PE”) on HR (increase), RMSSD (decrease), and RF (increase), based on classical cardiorespiratory effects observed with PE (Stanley et al., 2013; Menz et al., 2016; Mlynczak and Krysztofiak, 2019). We investigated this manipulation check hypothesis running three repeated-measures ANOVA, with condition (SPB vs. CON), time (PRE vs. POST) as within-subject independent variables and relaxation moment (“relax before PE” or “relax after PE”) as between-subject independent variables, with HR, RF, and RMSSD as dependent variables. Regarding RMSSD, in order to control for the potential effect of covariates, a linear mixed model analysis was conducted with age, gender, smoking status, BMI, and RF as covariates. The linear mixed model analysis allows to take into account time-varying covariates, in this case RF, which is not possible with the linear general model repeated-measures analysis module of SPSS. Given our hypothesis related to the manipulation check concerned only the main effect of time, we only report this result for clarity matters. Further, in order to ensure that changes are not due to a different amount of Burpees realized, the number of Burpees performed during the 5 min Burpees exercise will be checked via a repeated-measures ANOVA, with condition (SPB vs. CON) as within-subject independent variable and relaxation moment (“relax before PE” or “relax after PE”) as between-subject independent variable.

Regarding our main working hypothesis, we ran a repeated-measures ANOVA with condition (SPB vs. CON) as within-subject independent variable, and relaxation moment (“relax before PE” or “relax after PE”) as between-subject independent variable. Errors (error rate to incongruent stimuli, reflecting Stroop interference accuracy) and RTs (incongruent stimuli-congruent stimuli) were used as dependent variables for the Stroop interference, and RMSSD to infer CVA. Regarding errors in the Stroop task, we decided not to take into account errors made with congruent stimuli in the calculation, given they were none. Interactions were investigated with further *t*-tests either paired or independent according to the analysis, with Bonferroni correction regarding the significance level. Regarding RMSSD, similar to the previous analysis related to PE, a linear mixed model analysis was further conducted with age, gender, smoking status, BMI, and RF as covariates.

Finally, in case a significant effect of SPB on Stroop interference (errors and/or RTs) was found, potential mediation via RMSSD was performed via the PROCESS 3.3 dialog box developed by Hayes (2013). This custom dialog tests the total, direct, and indirect effect of an independent variable on a dependent variable through a proposed mediator and allows inferences regarding indirect effects using percentile bootstrap confidence intervals.

## Results

The full dataset is available in Supplementary Material.

### Perceived Stress Manipulation Check

**FIGURE 2:**
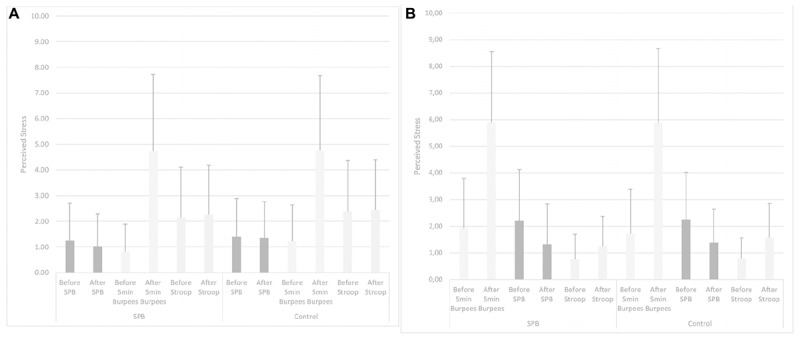
**(A,B)** Perceived stress in “Relax before PE.” SPB: slow-paced breathing; 5 min Burpees: 5 min Burpees exercise; Stroop: Color Word Stroop Test; “Relax before PE”: Experiment in which the relaxation moment (either slow-paced breathing or watching the TV documentary) took place before the 5 min Burpees exercise; “Relax after PE”: Experiment in which the relaxation moment (either slow-paced breathing or watching the TV documentary) took place after the 5 min Burpees exercise.

See Figures 2A,B for full descriptive statistics about PS.

#### Relaxation (SPB vs. Watching TV)

Greenhouse-Geisser correction was applied for the tests. Regarding PS before and after the relaxation moment, no main effect of condition was found, *F*(1,105) = 1.708, *p* = 0.187, partial η^2^ = 0.02. No interaction condition × relaxation moment was found, *F*(1,105) = 0.743, *p* = 0.391, partial η^2^ = 0. A main effect of time was found, *F*(1,105) = 17.808, *p* < 0.001, partial η^2^ = 0.15. PS before the relaxation was higher (*M* = 1.72; *SD* = 1.57) than after the relaxation (*M* = 1.45; *SD* = 1.26), *t*(106) = 3.111, *p* = 0.002; Cohen’s *d* = 0.19. An interaction effect time × relaxation moment was found, *F*(1,105) = 9.108, *p* = 0.003, partial η^2^ = 0.08. Further *post hoc t*-tests were run: they showed no significant difference for PS in “Relax before PE” being higher before (*M* = 1.32; *SD* = 1.31) than after (*M* = 1.18; *SD* = 1.19) the relaxation, *t*(59) = 1.359, *p* = 0.179; Cohen’s *d* = 0.12. A significant difference was found for PS being lower before relaxation in “relax before PE” (*M* = 0.90, *SD* = 0.95) in comparison to before relaxation in “relax after PE” (*M* = 2.22, *SD* = 1.73), *t*(46) = 5.426, *p* < 0.001; Cohen’s *d* = 0.96. PS was significantly lower after relaxation in “relax before PE” (*M* = 0.79, *SD* = 0.77) in comparison to in “relax after PE” (*M* = 1.79, *SD* = 1.29), *t*(46) = 6.795, *p* < 0.001; Cohen’s *d* = 0.95. In “relax after PE,” PS was significantly lower after (*M* = 1.79, *SD* = 1.29) than before (*M* = 2.22, *SD* = 1.73) relaxation, *t*(46) = 3.042, *p* = 0.004; Cohen’s *d* = 0.28. No interaction effect condition × time was found, *F*(1,105) = 0.302, *p* = 0.584, partial η^2^ = 0.0. No interaction effect condition × time × relaxation moment was found, *F*(1,105) = 0.178, *p* = 0.674, partial η^2^ = 0.0.

#### Five Minutes Burpees Exercise

Greenhouse-Geisser correction was applied for the tests. Regarding PS before and after the 5 min Burpees exercise, no main effect of condition was found, *F*(1,105) = 0.197, *p* = 0.658, partial η^2^ = 0. No interaction condition × relaxation moment was found, *F*(1,105) = 1.595, *p* = 0.209, partial η^2^ = 0.02. A main effect of time was found, *F*(1,105) = 181.695, *p* < 0.001, partial η^2^ = 0.63. PS after the 5 min Burpees exercise was higher (*M* = 5.24; *SD* = 2.73) than before the 5 min Burpees exercise (*M* = 1.37; *SD* = 1.41), *t*(106) = 13.556, *p* < 0.001; Cohen’s *d* = 1.79. No interaction effect time × relaxation moment was found, *F*(1,105) = 0.310, *p* = 0.579, partial η^2^ = 0. No interaction effect condition × time was found, *F*(1,105) = 0.159, *p* = 0.690, partial η^2^ = 0. No interaction effect condition × time × relaxation moment was found, *F*(1,105) = 1.995, *p* = 0.161, partial η^2^ = 0.02.

#### Stroop Task

Greenhouse-Geisser correction was applied for the tests. Regarding PS before and after the relaxation moment, no main effect of condition was found, *F*(1,105) = 2.102, *p* = 0.150, partial η^2^ = 0.02. No interaction condition × relaxation moment was found, *F*(1,105) = 0.020, *p* = 0.887, partial η^2^ = 0. A main effect of time was found, *F*(1,105) = 8.976, *p* = 0.003, partial η^2^ = 0.08. PS was higher after Stroop (*M* = 1.94; *SD* = 1.52) than before Stroop (*M* = 1.61; *SD* = 1.54), *t*(106) = 2.696, *p* < 0.001; Cohen’s *d* = 0.21. An interaction effect time × relaxation moment was found, *F*(1,105) = 4.963, *p* = 0.028, partial η^2^ = 0.05. In “relax before PE,” no difference in PS before (*M* = 2.27, *SD* = 1.70) and after Stroop (*M* = 2.36, *SD* = 1.69), *t*(59) = 0.500, *p* = 0.619; Cohen’s *d* = 0.05. Considering the time before the Stroop task, PS was higher in “relax before PE” (*M* = 2.02, *SD* = 1.69) in comparison to in “relax after PE” (*M* = 0.77, *SD* = 0.72); *t*(46) = 4.727, *p* < 0.001; Cohen’s *d* = 0.97. Considering the time after the Stroop task, PS was higher in “relax before PE” (*M* = 2.18, *SD* = 1.66) in comparison to “relax after PE” (*M* = 2.02, *SD* = 1.69), *t*(46) = 3.034, *p* = 0.004; Cohen’s *d* = 0.57. In “relax after PE,” PS was higher after the Stroop task (*M* = 1.40, *SD* = 1.06) than before the Stroop task (*M* = 0.77, *SD* = 0.72), *t*(46) = 4.664, *p* < 0.001; Cohen’s *d* = 0.69. No interaction effect condition × time was found, *F*(1,105) = 0.665, *p* = 0.417, partial η^2^ = 0.0. No interaction effect condition × time × relaxation moment was found, *F*(1,105) = 1.099, *p* = 0.297, partial η^2^ = 0.01.

### Five Minutes Burpees Exercise – Physiological Manipulation Check

**FIGURE 3:**
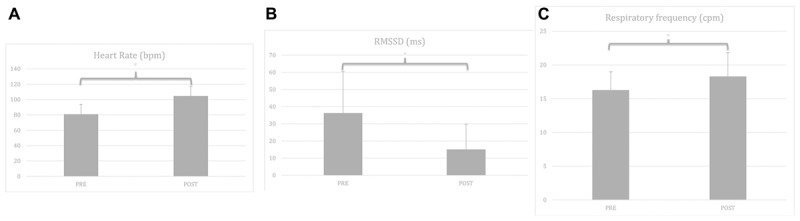
**(A)** Manipulation check for time effect with heart rate before and after the 5 min Burpees exercise. **(B)** Manipulation check for time effect with RMSSD before and after the 5 min Burpees exercise. **(C)** Manipulation check for time effect with respiratory frequency before and after the 5 min Burpees exercise. For the manipulation check of the 5 min Burpee exercise (PE – physical exertion), “PRE” refers to the 5 min resting measure realized before PE (so resting measure 1 for Exp. 1 “relax before PE” and resting measure 2 for Exp. 2 “relax after PE”), while “POST” refers to the 5 min resting measure realized after PE (so resting measure 2 for Exp. 1 “relax before PE” and resting measure 3 for Exp. 2. “relax after PE”). RMSSD: root mean square of successive differences.

Descriptive statistics for all physiological variables and all measurement points can be seen in Tables 1A,B, while descriptive statistics specifically related to the manipulation check of the 5 min Burpees exercise can be seen in Figures 3A–C.

**TABLE 1A:** Descriptive statistics physiological variables Exp. 1 “Relax before PE.”

	**SPB**								**Control**							
	**Resting 1 (before relax)**		**Resting 2 (after relax/PRE-PE)**		**Resting 3 (POST-PE/before Stroop)**		**Resting 4 (after Stroop)**		**Resting 1 (before relax)**		**Resting 2 (after relax/PRE-PE)**		**Resting 3 (POST-PE/before Stroop)**		**Resting 4 (after Stroop)**	
								
	***M***	***SD***	***M***	***SD***	***M***	***SD***	***M***	***SD***	***M***	***SD***	***M***	***SD***	***M***	***SD***	***M***	***SD***
Heart rate (bpm)	77.16	11.92	73.74	11.28	107.31	12.26	99.36	11.90	76.50	10.70	75.56	10.11	103.23	20.08	106.29	43.71
RMSSD (ms)	43.57	26.04	45.79	26.83	23.90	9.36	12.76	8.30	45.90	27.92	42.61	25.77	11.69	11.93	13.33	10.52
Respiratory frequency (cpm)	14.15	2.76	14.33	2.21	19.20	4.15	16.55	3.61	16.52	2.36	16.45	2.68	18.04	5.21	16.17	4.09

**TABLE 1B:** Descriptive statistics physiological variables Exp. 2 “Relax after PE.”

	**SPB**								**Control**							
		
	**Resting 1 (PRE-PE)**		**Resting 2 (POST-PE/before relax)**		**Resting 3 (after relax/before Stroop)**		**Resting 4 (after Stroop)**		**Resting 1 (PRE-PE)**		**Resting 2 (POST-PE/before relax)**		**Resting 3 (after relax/before Stroop)**		**Resting 4 (after Stroop)**	
								
	***M***	***SD***	***M***	***SD***	***M***	***SD***	***M***	***SD***	***M***	***SD***	***M***	***SD***	***M***	***SD***	***M***	***SD***
Heart rate (bpm)	89.67	13.50	107.12	14.10	92.72	11.18	95.61	12.20	87.85	14.91	100.70	13.25	103.23	20.08	89.39	10.14
RMSSD (ms)	25.38	18.82	17.72	23.90	24.22	16.19	17.44	11.39	27.12	27.84	21.54	26.91	17.38	10.90	19.76	11.23
Respiratory frequency (cpm)	17.23	3.59	17.31	4.39	11.20	3.20	12.50	3.02	17.58	3.35	18.47	3.59	15.06	3.18	13.97	3.05

*bpm: beats per minute; ms: milliseconds; cpm: cycles per minute; relax: slow-paced breathing exercise or watching TV neutral documentary; PE: physical exertion (5 min Burpees exercise); SPB: slow-paced breathing; RMSSD: root mean square of successive differences. For the manipulation check of the Burpee exercise, “PRE” refers to the 5 min resting measure realized before PE (so resting measure 1 for Exp. 1 “relax before PE” and resting measure 2 for Exp. 2 “relax after PE”). While “POST” refers to the 5 min resting measure realized after PE (so resting measure 2 for Exp. 1 “relax before PE” and resting measure 3 for Exp. 2 “relax after PE”).*

#### Heart Rate

Regarding HR, a main effect of time was found, *F*(1,105) = 344.906, *p* < 0.001, partial η^2^ = 0.69; with HR being higher after PE (*M* = 103.93; *SD* = 13.46) in comparison to before PE (*M* = 80.85, *SD* = 12.95).

#### RMSSD

Regarding RMSSD, a repeated-measures ANOVA was first ran. A main effect of time was found, *F*(1,105) = 85.517, *p* < 0.001, partial η^2^ = 0.40, with RMSSD being lower after PE (*M* = 13.43; *SD* = 12.53) than before PE (*M* = 24.87; *SD* = 24.23). A linear mixed model analysis was then ran to investigate whether covariates (RF, age, gender, smoking status, BMI) were affecting the results. Relaxation moment, condition, time, and all covariates were entered as fixed effects, including intercepts, with an unstructured repeated covariance type resulting in the best model fit, with – 2 Restricted Log Likelihood = 214.341. No random effects (slopes nor intercepts) were found to improve significantly the model fit, consequently none were added to the model. Regarding the main effect of time, results remained significant within a linear mixed model analysis, *F*(1,106.956) = 158.644, *p* < 0.001, estimate of fixed effect = −0.45 (*SE* = 0.04), 95% CI = −0.52 to −0.38. From the covariates two were found to have a significant effect, RF, *F*(1,263.319) = 34.114, *p* < 0.001, estimate of fixed effect = −0.70 (*SE* = 0.12), 95% CI = −0.93 to −0.46; and BMI, *F*(1,101.267) = 4.074, *p* = 0.046; estimate of fixed effect = −0.02 (*SE* = 0.01), 95% CI = −0.04 to 0.

#### Respiratory Frequency

Regarding RF, a main effect of time was found, *F*(1,105) = 45.262, *p* < 0.001, partial η^2^ = 0.26, with RF higher after PE (*M* = 18.30, *SD* = 3.54) than before PE (*M* = 16.28, *SD* = 2.71).

#### Number of Burpees

Regarding the number of Burpees performed during PE (descriptive statistics in Table 2), no main effect of condition was found *F*(1,105) = 0.161, *p* = 0.689, partial η^2^ = 0; and no interaction effect was found with the moment of relaxation *F*(1,105) = 0.524, *p* = 0.471, partial η^2^ = 0.01.

**TABLE 2:** Descriptive statistics for the number of Burpees realized during the 5 min Burpees exercise.

	**Exp. 1 – RELAX BEFORE PE**				**Exp. 2 – RELAX AFTER PE**			
		
	**Slow paced breathing**		**Control**		**Slow paced breathing**		**Control**	
				
	***M***	***SD***	***M***	***SD***	***M***	***SD***	***M***	***SD***
Number of Burpees	64.27	11.54	63.00	11.64	63.60	11.69	63.19	12.20

*PE: physical exertion (5 min Burpees exercise).*

### Main Research Question – Stroop Task

Descriptive statistics related to the variables linked to our main research question with the Stroop task are displayed in Table 3. Regarding Stroop interference (RT), a repeated-measures ANOVA revealed no main effect of condition *F*(1,105) = 0.873, *p* = 0.352, partial η^2^ = 0. No interaction effect condition × relaxation moment was found, *F*(1,105) = 0.039, *p* = 0.843, partial η^2^ = 0. Relaxation moment had an overall effect on the Stroop interference, *F*(1,105) = 35.031, *p* < 0.001, partial η^2^ = 0.25, with a shorter Stroop interference found for “relax before PE” (*M* = 20,33; *SD* = 69.61) in comparison to “relax after PE” (*M* = 103.14; *SD* = 64.87).

**TABLE 3:** Descriptive statistics for the main working hypothesis.

	**Exp. 1 – RELAX BEFORE PE**				**Exp. 2 – RELAX AFTER PE**			
		
	**SPB**		**Control**		**SPB**		**Control**	
				
	***M***	***SD***	***M***	***SD***	***M***	***SD***	***M***	***SD***
Stroop interference (number of errors)	0.50	0.70	0.68	0.81	0.17	0.38	0.70	0.86
Stroop interference (reaction times, ms)	18.26	95.70	25.61	79.45	104.71	72.01	109.49	68.92
RMSSD (ms)	11.29	9.36	11.69	11.93	24.22	16.20	11.69	11.93
Respiratory frequency (cpm)	19.20	4.15	18.04	5.212	11.20	3.201	15.06	3.18

*ms: milliseconds; cpm: cycles per minute; PE: physical exertion (5 min Burpees exercise); Stroop interference (accuracy) represents the number of errors in response to incongruent stimuli; Stroop interference (reaction time): reaction time to incongruent stimuli – reaction time to congruent stimuli; RMSSD, root mean square of successive differences; and respiratory frequency correspond to the resting measure 3 taken before the Stroop test.*

Regarding Stroop interference (errors), we found a main effect for condition, *F*(1,105) = 22.584, *p* < 0.001, partial η^2^ = 0.17, with SPB (*M* = 0.36, *SD* = 0.60) being lower than CON (*M* = 0.69, *SD* = 0.82). No main relaxation moment effect was found, *F*(1,105) = 1.735, *p* = 0.191, partial η^2^ = 0.02. An interaction effect between condition and relaxation moment was found, *F*(1,105) = 5.364, *p* = 0.022, partial η^2^ = 0.04. Further *post hoc t*-tests were run: they showed a significant difference between SPB-“relax before PE” (*M* = 0.50; *SD* = 0.70) and SPB-“relax after PE” (*M* = 0.17; *SD* = 0.38), with *t* = 4.105, df = 46, *p* < 0.001, Cohen’s *d* = 0.60; a significant difference between SPB-“relax before PE” (*M* = 0.50, *SD* = 0.70) and CON-“relax before PE” (*M* = 0.68, *SD* = 0.81), with *t* = 2.647, df = 59, *p* = 0.010, Cohen’s *d* = 0.34; a significant difference between SPB-“relax after PE” (*M* = 0.17, *SD* = 0.38) and CON-“relax after PE” (*M* = 0.70, *SD* = 0.86), *t* = 3.658, df = 46, *p* < 0.001, Cohen’s *d* = 0.53. No significant differences were found between CON-“relax before PE” and CON-“relax after PE,” *t* = 0.643, df = 46, *p* = 0.523, Cohen’s *d* = 0.09.

Regarding RMSSD during the resting measure before starting the Stroop test: a main condition effect was found, *F*(1,105) = 5.841, *p* = 0.017, partial η^2^ = 0.05, with SPB (*M* = 16.97, *SD* = 14.28) being higher than CON (*M* = 14.19, *SD* = 11.79). No interaction effect between condition and relaxation moment was found, *F*(1,105) = 3.335, *p* = 0.071, partial η^2^ = 0.03. A main relaxation moment effect was found, *F*(1,105) = 28.870, *p* < 0.001, partial η^2^ = 0.22, with RMSSD being higher in “relax after PE” (*M* = 20.80, *SD* = 14.15) than in “relax before PE” (*M* = 11.49, *SD* = 10.69).

A linear mixed model analysis was then ran to investigate whether covariates (RF, age, sex, smoking status, BMI) were affecting the results. Relaxation moment, condition, and all covariates were entered as fixed effects, including intercepts, with an unstructured repeated covariance type resulting in the best model fit, with – 2 Restricted Log Likelihood = 129.122. No random effects (slopes nor intercepts) were found to improve significantly the model fit, consequently none were added to the model. No main effect of condition was found, *F*(1,108.835) = 0.039, *p* = 0.844. A main effect of relaxation moment was found, *F*(1,126.990) = 4.890, *p* = 0.029, estimate of fixed effect = 0.27 (*SE* = 0.12), 95% CI = 0.03 to 0.51. No interaction effect condition × relaxation moment was found, *F*(1,110.459) = 0.169, *p* = 0.682. From the covariates only RF was found to have a significant effect, *F*(1,143.946) = 16.814, *p* < 0.001, estimate of fixed effect = −0.47 (*SE* = 0.11), 95% CI = −0.70 to −0.24.

Finally, to test whether the effect of SPB on Stroop interference (errors) was mediated by RMSSD, the experimental group (coded SPB = 1; CON = 2) was entered as independent variable, Stroop interference (errors) was entered as dependent variable, and RMSSD was entered as mediator variable. Based on a 10,000 sampling rate, the results from bootstrapping revealed no significant indirect effect, 95% CI = −0.15 to 0.18. Rerunning the mediation analysis taking into account the covariates (RF, age, sex, smoking status, BMI) revealed no significant indirect effect, 95% CI = −0.03 to 0.10.

## Discussion

This research aimed to investigate the effects of SPB on the adaptation to psychological stress, and more specifically inhibition performance, after PE. At the subjective level, in line with our hypothesis, PS was increased during the 5 min Burpees exercise and the Stroop test, and decreased during the relaxation technique, however, no effect of condition was found. At the physiological level, in line with our hypothesis, our PE manipulation was successful in increasing HR, RF, and decreasing RMSSD. Our main hypothesis was partially validated, given SPB led to better Stroop interference accuracy (lower number of errors in response to incongruent stimuli), however, no differences were found regarding Stroop interference RT.

Concerning the subjective manipulation check, confirming our hypothesis, PE and CWST increased PS, while PS was decreased with relaxation. This means that PE and the Stroop task were perceived as taxing or exceeding the adaptive resources of the individual, while relaxation contributed to decreased PS (Lazarus and Folkman, 1984). However, contrary to our hypothesis based on the relaxing effects of SPB (Lehrer and Gevirtz, 2014), the effects of SPB on PS did not differ from watching the TV documentary. This could be linked to the fact that many people already use TV as means of relaxation (Conway and Rubin, 2016), and therefore associate it with an activity decreasing PS. Interestingly, the increase in PS was much higher during the 5 min Burpees exercise (partial η^2^ = 0.63) in comparison to the Stroop task (partial η^2^ = 0.08). This might be due to the fact the 5 min Burpees exercise was perceived as particularly exhausting by the participants.

Concerning the physiological manipulation check of the 5 min Burpees exercise, we focused specifically on the effects involving time, given we are looking for physiological changes between the resting measures before and after the Burpees. HR and RF increased overall during the 5 min Burpees exercise, while RMSSD decreased (even after controlling for RF and other covariates). Our results cannot be directly compared to previous research, given very few studies have investigated Burpees so far, and when they did it was always as part as a more global training program involving a large range of physical exercises (McRae et al., 2012; Emberts et al., 2013; Abbes et al., 2018; Sperlich et al., 2018). However, these findings would be in line with typical cardiorespiratory adaptations to PE (Stanley et al., 2013; Menz et al., 2016; Mlynczak and Krysztofiak, 2019). The number of Burpees achieved did not differ across conditions or across relaxation moment. We should note that in the 5 min Burpees exercise, the realization of the Burpees was paced for the participants, meaning they could not go faster, even if they felt that they were able to do so. Further research may investigate whether giving the instruction to perform as many Burpees as possible during 5 min (i.e., maximal performance) may have provided different results, helping to understand whether SPB influences physical performance.

Regarding our main hypothesis linked to Stroop interference errors and RTs, Stroop interference errors decreased with SPB, while no change were observed for Stroop RTs, therefore our hypothesis was only partially validated. According to the literature, with the CWST inhibition is primarily reflected by the number of errors (accuracy) made with incongruent stimuli (McDowd et al., 1995), given it shows a typical illustration of inhibition failure. This is in line with the interpretation that Stroop interference accuracy represents as well an index of the ability to maintain the task’s goal temporarily in a highly retrievable state (Kane and Engle, 2003), given making a mistake linked to incongruent stimuli means that we were temporarily not successful in the aim of the task. A follow-up analysis of the condition × relaxation moment interaction shows that if Stroop interference errors were overall lower in the SPB condition in comparison to the CON condition, Stroop interference errors were lower when SPB was realized after PE than before PE. This may be explained by the time proximity of SPB to the realization of the Stroop test, and also by the fact that the physiological changes induced by PE may have influenced the effects of SPB realized beforehand.

Given no differences were observed with Stroop interference RTs, we have to conclude that SPB did not help to reduce the processing time of incongruent stimuli. Even if Stroop interference RTs are not the main marker of inhibition obtained with the Stroop test, RTs may still help us to understand mechanisms related to inhibition processing (Scarpina and Tagini, 2017). This may be linked to the fact SPB induces a general decrease of general physiological arousal (Lehrer and Gevirtz, 2014), which we also observed in this research in experiment 1 (“relax before PE”), where participants had a significantly lower HR and higher RMSSD after performing SPB in comparison to CON. The fact that physiological arousal plays a role in the Stroop interference RTs is also confirmed by our data, given Stroop interference RTs are much lower right after PE (in “relax before PE”), than 17 min later after performing SPB or CON (in “relax after PE”), with a large effect size (partial η^2^ = 25), which is in line with previous research (Cooper et al., 2016). Cooper et al. (2016) found that RTs on the complex level (incongruent condition) of the Stroop were quicker (in comparison to RTs obtained before exercise) immediately following the sprint-based exercise, but did not differ after 45 min; while RTs on the simple level (congruent condition) of the Stroop were quicker 45 min following sprint-based exercise (Cooper et al., 2016), but did not differ from the rest condition right after exercise. It could be speculated that this difference may be due to changes in brain-derived neurotrophic factor (Griffin et al., 2011) and brain activation in the left dorsolateral prefrontal cortex (Yanagisawa et al., 2010), given these mechanisms were previously identified as playing a role in improving Stroop interference RTs after exercise, however, this should be investigated in further research. To sum up, we found that SPB was effective in improving Stroop interference accuracy (decreasing the number of errors to incongruent stimuli) but not RTs.

Root mean square of successive differences in the resting measure before the Stroop task was found to be higher in the SPB condition than in the CON condition, which would mean that the increase in vagal afferent activity assumed by the resonance model (Lehrer and Gevirtz, 2014) can also be observed in vagal efferent activity (CVA). However, this main effect of condition disappears when integrating covariates to the analyses, and in particular RF, given a main effect of RF on RMSSD was found. Given respiratory parameters (Hirsch and Bishop, 1981; Brown et al., 1993; Houtveen et al., 2002), and in particular RF (Hirsch and Bishop, 1981) were found to influence HRV, some authors recommend to correct HRV variables reflecting CVA for respiration in order to accurately capture CVA (e.g., Grossman et al., 1991; Grossman, 1992). However, another stream of research assumes a common neural basis for HRV and respiration, and regards a routine control of HRV for respiration problematic, given it would remove variability associated with neural control over the heartbeat, and therefore some of the variance playing a crucial role in HRV would be artificially removed, which would then not reflect normal physiology (Larsen et al., 2010; Thayer et al., 2011; Lewis et al., 2012; Dick et al., 2014). For further clarification whether SPB does increase CVA, future research should consider manipulating parasympathetic nervous activity via pharmacological blockade, for example with atropine (Lahiri et al., 2008), a parasympatholytic agent that inhibits the action of acetylcholine, the main neurotransmitter of the parasympathetic nervous system, by competitively blocking muscarine receptors (Clementi and Weber-Schöndorfer, 2015). Previous research (Du Plooy and Venter, 1995) investigating deep breathing (three deep inspirations and expirations) and HRV with atropine injection showed that RMSSD was increased during deep breathing, however, this increase was canceled by atropine, which would suggest that this increase in RMSSD was vagally driven. These findings should however be replicated with the SPB exercise used in our study in order to clarify the effects of 15 min SPB on CVA.

Finally, a mediation analysis indicates that RMSSD did not mediate the effects of SPB on Stroop interference accuracy performance. This finding is not in line with the neurovisceral integration model (Thayer et al., 2009; Smith et al., 2017), which assumes that a higher CVA is linked to higher executive performance. These results are also contrary to what was found in Albinet et al. (2016), where improvements in Stroop interference (accuracy) following a 5-month aquaerobics program were correlated to increases in RMSSD, although no mediation analysis was performed. This may suggest that other mechanisms than CVA may underlie SPB effects. For example, the high amplitude oscillations in HR provoked by SPB were recently suggested to enhance the functional connectivity in brain networks associated with emotion regulation (Mather and Thayer, 2018). Future research has to clarify whether those high amplitude oscillations in HR due to SPB also provoke changes in the functional connectivity of brain networks associated with executive (inhibitory) functioning.

The strengths of this research were conducting two experiments to thoroughly investigate the effects of SPB on the adaptation to psychological stress, specifically inhibition, following PE; as well as the use of physiological measurements (HRV) to enable the investigation of potential underlying mechanisms. However, our research is not without limitation. Firstly, the participants’ sporting background (e.g., fitness level, sport experience) was not assessed. Secondly, our sample was comprised of sport students, consequently future research has to investigate whether our findings would replicate to other non-athletic samples. Thirdly, no color perception test was conducted, participants only stated if they were color-blind, however, they were only allowed to continue the experiment if they were successful during the CWST familiarization phase. Fourth, the determination of the 70% of the 5 min Burpees exercise during the pilot study was based on subjective evaluation with the Borg Scale of Perceived Exertion (Borg, 1982), and further research should also measure physiological parameters during the 5 min Burpees exercise. Fifth, due to technical problems our final sample comprised 107 participants instead of the 120 originally planned, and future studies should ensure to achieve the necessary sample size to detect whether CVA mediates executive performance following SPB, to rule out the possibility that our study was underpowered to find a mediation effect. Sixth, in order to shed more light on the underlying physiological mechanisms, other parameters should be investigated, such as gaseous exchange and brain activity for example. Future research may also consider the use of biofeedback to help the participants visualize the effects of SPB on their HRV and CVA.

## Conclusion

The aim of this research was to investigate the role of a relaxation technique, SPB, on the adaptation to psychological stress, investigated with a measure of inhibition, following PE. Two experiments were conducted within this research. In Experiment 1, SPB (or the TV watching control condition) was realized after PE, and in Experiment 2, SPB (or the TV watching control condition) was realized before PE. Our findings showed that SPB was able to improve inhibition after PE via improving Stroop interference accuracy, meaning that participants made less errors overall after having performed SPB (either before or after PE), and the effects were stronger when SPB was performed after PE. However, SPB was not found to impact Stroop interference RTs, meaning that participants were not faster in their ability to inhibit incongruent stimuli. The applied implications of these findings are quite interesting, given athletes can consider using SPB as a quick fix to address inhibition failures. SPB may therefore help to address choking under pressure, which can be triggered by inhibition failures according to the distraction account (Roberts et al., 2017). Finally, the use of SPB could be investigated in many domains where inhibition failures would lead to unwanted behavior with serious consequences, for example police officers shooting or medical doctors operating.

## Data Availability

All datasets generated for this study are included in the manuscript and the Supplementary Files.

## Ethics Statement

This study was approved by the Ethics Committee of the German Sport University Cologne (N° 175/2016).

## Author Contributions

SL, TL, and FD contributed to conceiving the design of the study. TL lead the data collection, with the help of SL. SL realized the statistical analysis with the help of TH and UB, and wrote the first draft of the manuscript. EM provided the critical comments to improve the manuscript. TH, UB, and FD suggested the final adjustments on the manuscript. All authors agreed on the final version of the manuscript.

## Conflict of Interest Statement

The authors declare that the research was conducted in the absence of any commercial or financial relationships that could be construed as a potential conflict of interest.

## Footnotes

1 No precise *a posteriori* power calculation could be performed, given exact descriptive statistics related to Stroop accuracy could not be extracted from the study.

2 https://www.youtube.com/watch?v=X60BcsO_wE (retrieved on April 3, 2019).

3 https://www.millisecond.com/download/library/stroop/ (retrieved on April 3, 2019).

4 https://www.youtube.com/watch?v=HDfvWrGUkC8 (retrieved on April 3, 2019).

## References

[B1] Abbes Z. Chamari K. Mujika I. Tabben M. Bibi K. W. Hussein A. M. (2018). Do Thirty-second post-activation potentiation exercises improve the 50-m freestyle sprint performance in adolescent swimmers? *Front. Physiol.* 9:1464. 10.3389/fphys.2018.01464 30459632 PMC6232934

[B2] Albinet C. T. Abou-Dest A. Andre N. Audiffren M. (2016). Executive functions improvement following a 5-month aquaerobics program in older adults: role of cardiac vagal control in inhibition performance. *Biol. Psychol.* 115 69–77. 10.1016/j.biopsycho.2016.01.010 26812613

[B3] Allen B. Friedman B. H. (2012). Positive emotion reduces dyspnea during slow paced breathing. *Psychophysiology* 49 690–696. 10.1111/j.1469-8986.2011.01344.x 22292794

[B4] Alves C. R. R. Gualano B. Takao P. P. Avakian P. Fernandes R. M. Morine D. (2012). Effects of acute physical exercise on executive functions: a comparison between aerobic and strength exercise. *J. Sport Exerc. Psychol.* 34 539–549. 10.1123/jsep.34.4.539 22889693

[B5] Archer J. A. Lee A. Qiu A. Annabel Chen S. H. (2018). Functional connectivity of resting-state, working memory and inhibition networks in perceived stress. *Neurobiol. Stress* 8 186–201. 10.1016/j.ynstr.2017.01.002 29888313 PMC5991324

[B6] Arnsten A. F. (2009). Stress signalling pathways that impair prefrontal cortex structure and function. *Nat. Rev. Neurosci.* 10 410–422. 10.1038/nrn2648 19455173 PMC2907136

[B7] Aubert A. E. Seps B. Beckers F. (2003). Heart rate variability in athletes. *Sports Med.* 33 889–919. 10.2165/00007256-200333120-3 12974657

[B8] Benarroch E. E. (1993). The central autonomic network: functional organization, dysfunction, and perspective. *Mayo Clin. Proc.* 68 988–1001. 10.1016/s0025-6196(12)62272-1 8412366

[B9] Berntson G. G. Bigger J. T. Eckberg D. L. Grossman P. Kaufmann P. G. Malik M. (1997). Heart rate variability: origins, methods, and interpretive caveats. *Psychophysiology* 34 623–648. 10.1111/j.1469-8986.1997.tb02140.x 9401419

[B10] Borg G. A. (1982). Psychophysical bases of perceived exertion. *Med. Sci. Sports Exerc.* 14 377–381. 7154893

[B11] Brown T. E. Beightol L. A. Koh J. Eckberg D. L. (1993). Important influence of respiration on human R-R interval power spectra is largely ignored. *J. Appl. Physiol.* 75 2310–2317. 10.1152/jappl.1993.75.5.2310 8307890

[B12] Brugnera A. Zarbo C. Tarvainen M. P. Marchettini P. Adorni R. Compare A. (2018). Heart rate variability during acute psychosocial stress: a randomized cross-over trial of verbal and non-verbal laboratory stressors. *Int. J. Psychophysiol.* 127 17–25. 10.1016/j.ijpsycho.2018.02.016 29501671

[B13] Burpee R. H. (1940). *Seven Quickly Administered Tests of Physical Capacity and Their Use in Detecting Physical Incapacity for Motor Activity in Men and Boys.* New York, NY: Columbia University.

[B14] Chang Y.-K. Chi L. Etnier J. L. Wang C.-C. Chu C.-H. Zhou C. (2014). Effect of acute aerobic exercise on cognitive performance: role of cardiovascular fitness. *Psychol. Sport Exerc.* 15 464–470. 10.1016/j.psychsport.2014.04.007

[B15] Clementi M. Weber-Schöndorfer C. (2015). “Gastro-intestinal medications, hypolipidemic agents and spasmolytics,” in *Drugs During Pregnancy and Lactation*, eds Schaefer C. Peters P. Miller R. K. (New York, NY: Academic Press), 93–113. 10.1016/b978-0-12-408078-2.00006-8

[B16] Conway J. C. Rubin A. M. (2016). Psychological predictors of television viewing motivation. *Commun. Res.* 18 443–463. 10.1177/009365091018004001

[B17] Cooper S. B. Bandelow S. Nute M. L. Dring K. J. Stannard R. L. Morris J. G. (2016). Sprint-based exercise and cognitive function in adolescents. *Prevent. Med. Rep.* 4 155–161. 10.1016/j.pmedr.2016.06.004 27413677 PMC4929070

[B18] Diamond A. (2013). Executive functions. *Annu. Rev. Psychol.* 64 135–168. 10.1146/annurev-psych-113011-143750 23020641 PMC4084861

[B19] Dick T. E. Hsieh Y. H. Dhingra R. R. Baekey D. M. Galan R. F. Wehrwein E. (2014). Cardiorespiratory coupling: common rhythms in cardiac, sympathetic, and respiratory activities. *Prog. Brain Res.* 209 191–205. 10.1016/B978-0-444-63274-6.00010-2 24746049 PMC4052709

[B20] Dresler T. Meriau K. Heekeren H. R. Van Der Meer E. (2009). Emotional Stroop task: effect of word arousal and subject anxiety on emotional interference. *Psychol. Res.* 73 364–371. 10.1007/s00426-008-0154-6 18636272

[B21] Du Plooy W. J. Venter C. P. (1995). The effect of atropine on parasympathetic control of respiratory sinus arrhythmia in two ethnic groups. *J. Clin. Pharmacol.* 35 244–249. 10.1002/j.1552-4604.1995.tb04054.x 7608312

[B22] Emberts T. Porcari J. Dobers-Tein S. Steffen J. Foster C. (2013). Exercise intensity and energy expenditure of a tabata workout. *J. Sports Sci. Med.* 12 612–613. 24137082 PMC3772611

[B23] Faul F. Erdfelder E. Lang A. G. Buchner A. (2007). G^∗^Power 3: a flexible statistical power analysis program for the social, behavioral, and biomedical sciences. *Behav. Res. Methods* 39 175–191. 10.3758/bf03193146 17695343

[B24] Folkard S. (1990). Circadian performance rhythms: some practical and theoretical implications. *Philos. Trans. R. Soc. B Biol. Sci.* 327 543–553. 10.1098/rstb.1990.0097 1970900

[B25] Goldsmith R. L. Bloomfield D. M. Rosenwinkel E. T. (2000). Exercise and autonomic function. *Coron. Artery Dis.* 11 129–135. 10.1097/00019501-200003000-7 10758814

[B26] Griffin E. W. Mullally S. Foley C. Warmington S. A. O’Mara S. M. Kelly A. M. (2011). Aerobic exercise improves hippocampal function and increases BDNF in the serum of young adult males. *Physiol. Behav.* 104 934–941. 10.1016/j.physbeh.2011.06.005 21722657

[B27] Grossman P. (1992). Respiratory and cardiac rhythms as windows to central and autonomic biobehavioral regulation: selection of window frames, keeping the panes clean and viewing the neural topography. *Biol. Psychol.* 34 131–161. 10.1016/0301-0511(92)90013-k 1467391

[B28] Grossman P. Karemaker J. Wieling W. (1991). Prediction of tonic parasympathetic cardiac control using respiratory sinus arrhythmia: the need for respiratory control. *Psychophysiology* 28 201–216. 10.1111/j.1469-8986.1991.tb00412.x 1946886

[B29] Guo Z. Chen R. Liu X. Zhao G. Zheng Y. Gong M. (2018). The impairing effects of mental fatigue on response inhibition: an ERP study. *PLoS One* 13:e0198206. 10.1371/journal.pone.0198206 29856827 PMC5983454

[B30] Hayes A. F. (2013). *Introduction to Mediation, Moderation, and Conditional Process Analysis.* New York, NY: The Guilford Press.

[B31] Hill L. K. Siebenbrock A. Sollers J. J. Thayer J. F. (2009). Are all measures created equal? Heart rate variability and respiration. *Biomed. Sci. Instrum.* 45 71–76. 19369742

[B32] Hirsch J. A. Bishop B. (1981). Respiratory sinus arrhythmia in humans: how breathing pattern modulates heart rate. *Am. J. Physiol.* 241 H620–H629. 7315987 10.1152/ajpheart.1981.241.4.H620

[B33] Hoshikawa Y. Yamamoto Y. (1997). Effects of Stroop color-word conflict test on the autonomic nervous system responses. *Am. J. Physiol.* 272 H1113–H1121. 10.1152/ajpheart.1997.272.3.H1113 9087583

[B34] Houtveen J. H. Rietveld S. de Geus E. J. (2002). Contribution of tonic vagal modulation of heart rate, central respiratory drive, respiratory depth, and respiratory frequency to respiratory sinus arrhythmia during mental stress and physical exercise. *Psychophysiology* 39 427–436. 10.1111/1469-8986.3940427 12212635

[B35] Iellamo F. (2001). Neural mechanisms of cardiovascular regulation during exercise. *Auton. Neurosci.* 90 66–75. 10.1016/s1566-0702(01)00269-7 11485294

[B36] Inquisit 5 [Computer Software] (2016). Available at: https://www.millisecond.com

[B37] Inzlicht M. Schmeichel B. J. Macrae C. N. (2014). Why self-control seems (but may not be) limited. *Trends Cogn. Sci.* 18 127–133. 10.1016/j.tics.2013.12.009 24439530

[B38] Johnsen B. H. Thayer J. F. Laberg J. C. Wormnes B. Raadal M. Skaret E. (2003). Attentional and physiological characteristics of patients with dental anxiety. *J. Anxiety Disord.* 17 75–87. 10.1016/s0887-6185(02)00178-0 12464290

[B39] Johnson L. Addamo P. K. Selva Raj I. Borkoles E. Wyckelsma V. Cyarto E. (2016). An Acute bout of exercise improves the cognitive performance of older adults. *J. Aging Phys. Act.* 24 591–598. 10.1123/japa.2015-2097 26964644

[B40] Kane M. J. Engle R. W. (2003). Working-memory capacity and the control of attention: the contributions of goal neglect, response competition, and task set to Stroop interference. *J. Exp. Psychol. Gen.* 132 47–70. 10.1037/0096-3445.132.1.47 12656297

[B41] Kashihara K. Maruyama T. Murota M. Nakahara Y. (2009). Positive effects of acute and moderate physical exercise on cognitive function. *J. Physiol. Anthropol.* 28 155–164. 10.2114/jpa2.28.155 19652447

[B42] Kotabe H. P. Hofmann W. (2015). On integrating the components of self-control. *Perspect. Psychol. Sci.* 10 618–638. 10.1177/1745691615593382 26386000

[B43] Kurzban R. Duckworth A. Kable J. W. Myers J. (2013). An opportunity cost model of subjective effort and task performance. *Behav. Brain Sci.* 36 661–679. 10.1017/S0140525X12003196 24304775 PMC3856320

[B44] Laborde S. Allen M. S. Gohring N. Dosseville F. (2017a). The effect of slow-paced breathing on stress management in adolescents with intellectual disability. *J. Intellect. Disabil. Res.* 61 560–567. 10.1111/jir.12350 27933677

[B45] Laborde S. Mosley E. Thayer J. F. (2017b). Heart rate variability and cardiac vagal tone in psychophysiological research - recommendations for experiment planning, data analysis, and data reporting. *Front. Physiol.* 8:213. 10.3389/fpsyg.2017.00213 28265249 PMC5316555

[B46] Laborde S. Dosseville F. Aloui A. Ben Saad H. Bertollo M. Bortoli L. (2018a). Convergent and construct validity and test-retest reliability of the Caen Chronotype Questionnaire in six languages. *Chronobiol. Int.* 35 1294–1304. 10.1080/07420528.2018.1475396 29873546

[B47] Laborde S. Mosley E. Mertgen A. (2018b). A unifying conceptual framework of factors associated to cardiac vagal control. *Heliyon* 4:e01002. 10.1016/j.heliyon.2018.e01002 30623126 PMC6313821

[B48] Laborde S. Mosley E. Ueberholz L. (2018c). Enhancing cardiac vagal activity: factors of interest for sport psychology. *Prog. Brain Res.* 240 71–92. 10.1016/bs.pbr.2018.09.002 30390842

[B49] Laborde S. Hosang T. Mosley E. Dosseville F. (2019). Influence of a 30-day slow paced breathing intervention compared to social media use on subjective sleep quality and cardiac vagal activity. *J. Clin. Med.* 8:193. 10.3390/jcm8020193 30736268 PMC6406675

[B50] Laborde S. Raab M. Kinrade N. P. (2014). Is the ability to keep your mind sharp under pressure reflected in your heart? Evidence for the neurophysiological bases of decision reinvestment. *Biol. Psychol.* 100C 34–42. 10.1016/j.biopsycho.2014.05.003 24859424

[B51] Lahiri M. K. Kannankeril P. J. Goldberger J. J. (2008). Assessment of autonomic function in cardiovascular disease: physiological basis and prognostic implications. *J. Am. Coll. Cardiol.* 51 1725–1733. 10.1016/j.jacc.2008.01.038 18452777

[B52] Lambourne K. Tomporowski P. (2010). The effect of exercise-induced arousal on cognitive task performance: a meta-regression analysis. *Brain Res.* 1341 12–24. 10.1016/j.brainres.2010.03.091 20381468

[B53] Larsen P. D. Tzeng Y. C. Sin P. Y. Galletly D. C. (2010). Respiratory sinus arrhythmia in conscious humans during spontaneous respiration. *Respir. Physiol. Neurobiol.* 174 111–118. 10.1016/j.resp.2010.04.021 20420940

[B54] Lautenbach F. Laborde S. Putman P. Angelidis A. Raab M. (2016). Attentional distraction by negative sports words in athletes under low- and high-pressure conditions: evidence from the sport emotional Stroop task. *Sport Exerc. Perform. Psychol.* 5 296–307. 10.1037/spy0000073

[B55] Lazarus R. S. Folkman S. (1984). *Stress, Appraisal and Coping.* New York, NY: Springer.

[B56] Lehrer P. M. Gevirtz R. (2014). Heart rate variability biofeedback: how and why does it work? *Front. Psychol.* 5:756. 10.3389/fpsyg.2014.00756 25101026 PMC4104929

[B57] Lehrer P. M. Vaschillo E. Lu S. E. Eckberg D. Vaschillo B. Scardella A. (2006). Heart rate variability biofeedback: effects of age on heart rate variability, baroreflex gain, and asthma. *Chest* 129 278–284. 10.1378/chest.129.2.278 16478842

[B58] Lesage F.-X. Berjot S. (2011). Validity of occupational stress assessment using a visual analogue scale. *Occup. Med.* 61 434–436. 10.1093/occmed/kqr037 21505089

[B59] Lewis G. F. Furman S. A. McCool M. F. Porges S. W. (2012). Statistical strategies to quantify respiratory sinus arrhythmia: are commonly used metrics equivalent? *Biol. Psychol.* 89 349–364. 10.1016/j.biopsycho.2011.11.009 22138367 PMC3269511

[B60] Malik M. (1996). Heart rate variability. Standards of measurement, physiological interpretation, and clinical use. Task force of the European society of cardiology and the North American society of pacing and electrophysiology. *Eur. Heart J.* 17 354–381. 8737210

[B61] Mather M. Thayer J. F. (2018). How heart rate variability affects emotion regulation brain networks. *Curr. Opin. Behav. Sci.* 19 98–104. 10.1016/j.cobeha.2017.12.017 29333483 PMC5761738

[B62] McDowd J. M. Oseas-Kreger D. M. Filion D. L. (1995). “Inhibitory processes in cognition and aging,” in *Interference and Inhibition in Cognition*, eds Dempster F. N. Brainerd C. J. (San Diego, CA: Academic Press), 363–400. 10.1016/b978-012208930-5/50012-x

[B63] McRae G. Payne A. Zelt J. G. Scribbans T. D. Jung M. E. Little J. P. (2012). Extremely low volume, whole-body aerobic-resistance training improves aerobic fitness and muscular endurance in females. *Appl. Physiol. Nutr. Metab.* 37 1124–1131. 10.1139/h2012-093 22994393

[B64] Menz V. Semsch M. Mosbach F. Burtscher M. (2016). Cardiorespiratory effects of one-legged high-intensity interval training in normoxia and hypoxia: a pilot study. *J. Sports Sci. Med.* 15 208–213. 27274656 PMC4879432

[B65] Michael S. Graham K. S. Davis G. M. O. (2017). Cardiac autonomic responses during exercise and post-exercise recovery using heart rate variability and systolic time intervals-a review. *Front. Physiol.* 8:301. 10.3389/fphys.2017.00301 28611675 PMC5447093

[B66] Miyake A. Friedman N. P. Emerson M. J. Witzki A. H. Howerter A. Wager T. D. (2000). The unity and diversity of executive functions and their contributions to complex “Frontal Lobe” tasks: a latent variable analysis. *Cogn. Psychol.* 41 49–100. 10.1006/cogp.1999.0734 10945922

[B67] Mlynczak M. Krysztofiak H. (2019). Cardiorespiratory temporal causal links and the differences by sport or lack thereof. *Front. Physiol.* 10:45. 10.3389/fphys.2019.00045 30804797 PMC6370652

[B68] Ogoh S. Tsukamoto H. Hirasawa A. Hasegawa H. Hirose N. Hashimoto T. (2014). The effect of changes in cerebral blood flow on cognitive function during exercise. *Physiol. Rep.* 2:e12163. 10.14814/phy2.12163 25263210 PMC4270220

[B69] Peruyero F. Zapata J. Pastor D. Cervello E. (2017). The Acute effects of exercise intensity on inhibitory cognitive control in adolescents. *Front. Psychol.* 8:921. 10.3389/fpsyg.2017.00921 28620337 PMC5450506

[B70] Ploughman M. (2008). Exercise is brain food: the effects of physical activity on cognitive function. *Dev. Neurorehabil.* 11 236–240. 10.1080/17518420801997007 18781504

[B71] Podstawski R. Kasietczuk B. Boraczyñski T. Boraczyñski M. Choszcz D. (2013). Relationship between BMI and endurance-strength abilities assessed by the 3 minute burpee test. *Int. J. Sports Sci. Coach.* 3 28–35. 10.5923/j.sports.20130301.06

[B72] Prinsloo G. E. Rauch H. G. Lambert M. I. Muench F. Noakes T. D. Derman W. E. (2011). The effect of short duration heart rate variability (HRV) biofeedback on cognitive performance during laboratory induced cognitive stress. *Appl. Cogn. Psychol.* 25 792–801. 10.1002/acp.1750

[B73] Putman P. Berling S. (2011). Cortisol acutely reduces selective attention for erotic words in healthy young men. *Psychoneuroendocrinology* 36 1407–1417. 10.1016/j.psyneuen.2011.03.015 21497444

[B74] Quintana D. S. Heathers J. A. (2014). Considerations in the assessment of heart rate variability in biobehavioral research. *Front. Physiol.* 5:805. 10.3389/fpsyg.2014.00805 25101047 PMC4106423

[B75] Roberts L. J. Jackson M. S. Grundy I. H. (2017). Choking under pressure: illuminating the role of distraction and self-focus. *Int. Rev. Sport Exerc. Psychol.* 12 325–355. 10.1080/1750984x.2017.1374432

[B76] Romero S. A. Minson C. T. Halliwill J. R. (2017). The cardiovascular system after exercise. *J. Appl. Psychol.* 122 925–932. 10.1152/japplphysiol.00802.2016 28153943 PMC5407206

[B77] Satish P. Muralikrishnan K. Balasubramanian K. Shanmugapriya (2015). Heart rate variability changes during stroop color and word test among genders. *Indian J. Physiol. Pharmacol.* 59 9–15. 26571978

[B78] Scarpina F. Tagini S. (2017). The Stroop color and word test. *Front. Psychol.* 8:557. 10.3389/fpsyg.2017.00557 28446889 PMC5388755

[B79] Schmit C. Brisswalter J. (2018). Executive functioning during prolonged exercise: a fatigue-based neurocognitive perspective. *Int. Rev. Sport Exerc. Psychol.* 10.1080/1750984X.2018.1483527

[B80] Schmit C. Davranche K. Easthope C. S. Colson S. S. Brisswalter J. Radel R. (2015). Pushing to the limits: the dynamics of cognitive control during exhausting exercise. *Neuropsychologia* 68 71–81. 10.1016/j.neuropsychologia.2015.01.006 25576908

[B81] Sherwood L. (2006). *Fundamentals of Physiology: A Human Perspective.* Belmont, CA: Brooks/Cole.

[B82] Smith R. Thayer J. F. Khalsa S. S. Lane R. D. (2017). The hierarchical basis of neurovisceral integration. *Neurosci. Biobehav. Rev.* 75 274–296. 10.1016/j.neubiorev.2017.02.003 28188890

[B83] Spangler D. P. Friedman B. H. (2017). A Little goes a long way: low working memory load is associated with optimal distractor inhibition and increased vagal control under anxiety. *Front. Hum. Neurosci.* 11:43. 10.3389/fnhum.2017.00043 28217091 PMC5289964

[B84] Spangler D. P. Gamble K. R. McGinley J. J. Thayer J. F. Brooks J. R. (2018). Intra-individual variability in vagal control is associated with response inhibition under stress. *Front. Hum. Neurosci.* 12:475. 10.3389/fnhum.2018.00475 30542274 PMC6277930

[B85] Sperlich B. Hahn L. S. Edel A. Behr T. Helmprobst J. Leppich R. (2018). A 4-Week intervention involving mobile-based daily 6-minute micro-sessions of functional high-intensity circuit training improves strength and quality of life, but not cardio-respiratory fitness of young untrained adults. *Front. Physiol.* 9:423. 10.3389/fphys.2018.00423 29867519 PMC5954292

[B86] Stanley J. Peake J. M. Buchheit M. (2013). Cardiac parasympathetic reactivation following exercise: implications for training prescription. *Sports Med.* 43 1259–1277. 10.1007/s40279-013-0083-4 23912805

[B87] Strauss-Blasche G. Moser M. Voica M. McLeod D. R. Klammer N. Marktl W. (2000). Relative timing of inspiration and expiration affects respiratory sinus arrhythmia. *Clin. Exp. Pharmacol. Physiol.* 27 601–606. 10.1046/j.1440-1681.2000.03306.x 10901389

[B88] Stroop J. R. (1935). Studies of interference in serial verbal reactions. *J. Exp. Psychol.* 18 643–662. 10.1037/h0054651

[B89] Subramanya P. Telles S. (2015). Performance in the stroop task and simultaneously recorded heart rate variability before and after meditation, supine rest and no-intervention. *Int. J. Brain Cogn. Sci.* 4 8–14.

[B90] Tanaka M. Ishii A. Watanabe Y. (2013). Neural correlates of central inhibition during physical fatigue. *PLoS One* 8:e70949. 10.1371/journal.pone.0070949 23923034 PMC3724771

[B91] Tarvainen M. P. Niskanen J. P. Lipponen J. A. Ranta-Aho P. O. Karjalainen P. A. (2014). Kubios HRV–heart rate variability analysis software. *Comput. Methods Progr. Biomed.* 113 210–220. 10.1016/j.cmpb.2013.07.024 24054542

[B92] Thayer J. F. Hansen A. L. Saus-Rose E. Johnsen B. H. (2009). Heart rate variability, prefrontal neural function, and cognitive performance: the neurovisceral integration perspective on self-regulation, adaptation, and health. *Ann. Behav. Med.* 37 141–153. 10.1007/s12160-009-9101-z 19424767

[B93] Thayer J. F. Loerbroks A. Sternberg E. M. (2011). Inflammation and cardiorespiratory control: the role of the vagus nerve. *Respir. Physiol. Neurobiol.* 178 387–394. 10.1016/j.resp.2011.05.016 21642019

[B94] Tortora G. J. Derrickson B. H. (2014). *Principles of Anatomy and Physiology.* Hoboken, NJ: John Wiley & Sons, Inc.

[B95] van Eekelen A. P. Houtveen J. H. Kerkhof G. A. (2004). Circadian variation in cardiac autonomic activity: reactivity measurements to different types of stressors. *Chronobiol. Int.* 21 107–129. 10.1081/cbi-120027983 15129827

[B96] Vaschillo E. G. Lehrer P. Rishe N. Konstantinov M. (2002). Heart rate variability biofeedback as a method for assessing baroreflex function: a preliminary study of resonance in the cardiovascular system. *Appl. Psychophysiol. Biofeedback* 27 1–27. 12001882 10.1023/a:1014587304314

[B97] Vaschillo E. G. Vaschillo B. Lehrer P. M. (2006). Characteristics of resonance in heart rate variability stimulated by biofeedback. *Appl. Psychophysiol. Biofeedback* 31 129–142. 10.1007/s10484-006-9009-3 16838124

[B98] Vazan R. Filcikova D. Mravec B. (2017). Effect of the stroop test performed in supine position on the heart rate variability in both genders. *Auton. Neurosci. Basic Clin.* 208 156–160. 10.1016/j.autneu.2017.10.009 29108935

[B99] Vincent C. M. Hall P. A. (2017). Cognitive effects of a 30-min aerobic exercise bout on adults with overweight/obesity and type 2 diabetes. *Obes. Sci. Pract.* 3 289–297. 10.1002/osp4.112 29071105 PMC5598020

[B100] Wendt J. Neubert J. Koenig J. Thayer J. F. Hamm A. O. (2015). Resting heart rate variability is associated with inhibition of conditioned fear. *Psychophysiology* 52 1161–1166. 10.1111/psyp.12456 26095980

[B101] Winsley R. (2002). Acute and chronic effects of exercise on heart rate variability in adults and children: a review. *Pediatr. Exerc. Sci.* 14 328–344. 10.1123/pes.14.4.328

[B102] Yanagisawa H. Dan I. Tsuzuki D. Kato M. Okamoto M. Kyutoku Y. (2010). Acute moderate exercise elicits increased dorsolateral prefrontal activation and improves cognitive performance with stroop test. *Neuroimage* 50 1702–1710. 10.1016/j.neuroimage.2009.12.023 20006719

[B103] Zeki Al Hazzouri A. Elfassy T. Carnethon M. R. Lloyd-Jones D. M. Yaffe K. (2017). Heart rate variability and cognitive function in middle-age adults: the coronary artery risk development in young adults. *Am. J. Hypert.* 31 27–34. 10.1093/ajh/hpx125 28985245 PMC5861561

